# Serum neurofilament light chain protein is a measure of disease intensity in frontotemporal dementia

**DOI:** 10.1212/WNL.0000000000003154

**Published:** 2016-09-27

**Authors:** Jonathan D. Rohrer, Ione O.C. Woollacott, Katrina M. Dick, Emilie Brotherhood, Elizabeth Gordon, Alexander Fellows, Jamie Toombs, Ronald Druyeh, M. Jorge Cardoso, Sebastien Ourselin, Jennifer M. Nicholas, Niklas Norgren, Simon Mead, Ulf Andreasson, Kaj Blennow, Jonathan M. Schott, Nick C. Fox, Jason D. Warren, Henrik Zetterberg

**Affiliations:** From the Dementia Research Centre (J.D.R., I.O.C.W., K.M.D., E.B., E.G., A.F., M.J.C., S.O., J.M.N., J.M.S., N.C.F., J.D.W.), MRC Prion Unit (S.M., R.D.), Department of Neurodegenerative Disease, and Department of Molecular Neuroscience (J.T., H.Z.), UCL Institute of Neurology, Queen Square; Centre for Medical Image Computing (J.M.C., S.O.), University College London; Department of Medical Statistics (J.M.N.), London School of Hygiene and Tropical Medicine, UK; UmanDiagnostics (N.N.), Umeå; and Clinical Neurochemistry Laboratory (U.A., K.B., H.Z.), Department of Psychiatry and Neurochemistry, Institute of Neuroscience and Physiology, Sahlgrenska Academy at the University of Gothenburg, Mölndal, Sweden.

## Abstract

**Objective:**

To investigate serum neurofilament light chain (NfL) concentrations in frontotemporal dementia (FTD) and to see whether they are associated with the severity of disease.

**Methods:**

Serum samples were collected from 74 participants (34 with behavioral variant FTD [bvFTD], 3 with FTD and motor neuron disease and 37 with primary progressive aphasia [PPA]) and 28 healthy controls. Twenty-four of the FTD participants carried a pathogenic mutation in *C9orf72* (9), microtubule-associated protein tau (*MAPT*; 11), or progranulin (*GRN*; 4). Serum NfL concentrations were determined with the NF-Light kit transferred onto the single-molecule array platform and compared between FTD and healthy controls and between the FTD clinical and genetic subtypes. We also assessed the relationship between NfL concentrations and measures of cognition and brain volume.

**Results:**

Serum NfL concentrations were higher in patients with FTD overall (mean 77.9 pg/mL [SD 51.3 pg/mL]) than controls (19.6 pg/mL [SD 8.2 pg/mL]; *p* < 0.001). Concentrations were also significantly higher in bvFTD (57.8 pg/mL [SD 33.1 pg/mL]) and both the semantic and nonfluent variants of PPA (95.9 and 82.5 pg/mL [SD 33.0 and 33.8 pg/mL], respectively) compared with controls and in semantic variant PPA compared with logopenic variant PPA. Concentrations were significantly higher than controls in both the *C9orf72* and *MAPT* subgroups (79.2 and 40.5 pg/mL [SD 48.2 and 20.9 pg/mL], respectively) with a trend to a higher level in the *GRN* subgroup (138.5 pg/mL [SD 103.3 pg/mL). However, there was variability within all groups. Serum concentrations correlated particularly with frontal lobe atrophy rate (*r* = 0.53, *p* = 0.003).

**Conclusions:**

Increased serum NfL concentrations are seen in FTD but show wide variability within each clinical and genetic group. Higher concentrations may reflect the intensity of the disease in FTD and are associated with more rapid atrophy of the frontal lobes.

Frontotemporal dementia (FTD) is a common cause of early-onset dementia.^1^ Clinically, patients present with either changes in personality (behavioral variant FTD [bvFTD]) or impaired language (primary progressive aphasia [PPA]), although overlap with motor neuron disease (FTD-MND) is not uncommon.^1^ FTD has an autosomal dominant genetic cause in around a quarter of people, with mutations in the progranulin (*GRN*), chromosome 9 open reading frame 72 (*C9orf72*), and microtubule-associated protein tau (*MAPT*) genes being commonest.^2^

Few fluid biomarkers have been investigated in FTD, although there have now been a number of studies of neurofilament concentration in the CSF.^3–11^ Higher neurofilament light chain (NfL) levels are believed to represent axonal degeneration,^12,13^ and while early studies showed variability in CSF concentrations in FTD,^4–10^ a more recent study has suggested that CSF NfL levels correlate with disease severity.^11^

There is considerable interest in developing blood-based biomarkers because of their convenience and higher acceptability relative to CSF. NfL can be measured in serum with standard immunoassay formats,^14^ but those based on ELISA or electrochemiluminescence methods lack the analytical sensitivity to measure low levels. For this reason, we developed an immunoassay based on the single-molecule array (Simoa) technique^15^ that allows quantification down to subfemtomolar concentrations (<1 pg/mL) of the analyte and is 25-fold more sensitive than the previous electrochemiluminescence-based method.^16^ Using this assay, we aimed to investigate serum NfL concentrations in FTD. Our hypotheses were that serum NfL concentration would be elevated in patients with FTD compared with healthy controls, that concentrations would vary between FTD subgroups, and that increased serum NfL levels would reflect the disease intensity or rate of progression.

## METHODS

Seventy-four participants were consecutively recruited from the University College London FTD study: 34 participants with bvFTD according to Rascovsky criteria,^17^ 3 participants with FTD-MND,^18^ and 37 participants with PPA according to the Gorno-Tempini criteria.^19^ Of the 37 PPA participants, 13 had the nonfluent variant (nfvPPA), 10 had the semantic variant (svPPA), 7 had the logopenic variant (lvPPA), and 7 did not fit criteria for any of the 3 variants (PPA-NOS, not otherwise specified). We did not include patients fulfilling criteria for lvPPA in the overall FTD analysis because they are likely to have underlying Alzheimer disease pathologically.^1,2^ Data were compared with data from 28 healthy control participants matched for age and sex who had been collected as part of a study of neurodegenerative disease (table 1). Twenty-four of the FTD participants carried a pathogenic mutation: 9 with an expansion in *C9orf72* (8 with bvFTD, 1 with nfvPPA), 11 with an *MAPT* mutation (all with bvFTD), and 4 with a *GRN* mutation (1 with bvFTD, 1 with nfvPPA, and 2 with PPA-NOS). No mutations were found in the other participants. No significant differences were noted in age or sex between any of the groups, and no significant difference in disease duration was seen between the clinical or genetic FTD subgroups.

**Table 1:**
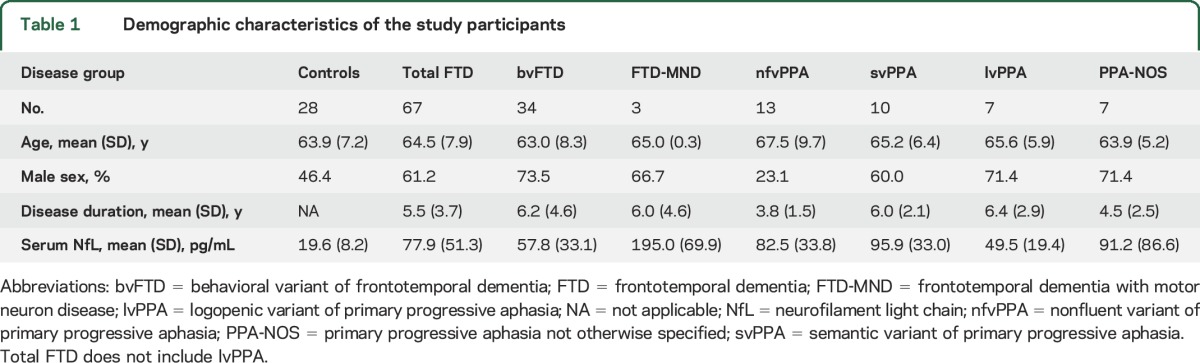
Demographic characteristics of the study participants

### Standard protocol approvals, registrations, and patient consents.

Approval for the study was obtained from the local ethics committee, and all participants provided written consent to take part.

### Measurement of NfL concentrations.

Serum samples were collected from each of the participants and then processed, divided into aliquots, and frozen at −80°C according to standardized procedures. Serum NfL concentrations were measured with the NF-Light assay from UmanDiagnostics (Umeå, Sweden) and transferred onto the Simoa platform with a home-brew kit (Quanterix Corp, Boston, MA). Detailed instructions can be found in the Simoa Homebrew Assay Development Guide (Quanterix). In short, paramagnetic carboxylated beads (catalog no. 100451, Quanterix) were activated by adding 5% (vol/vol) 10 mg/mL 1-ethyl-3-(3-dimethylaminopropyl) carbodiimide (catalog no. 100022, Quanterix) to a magnetic beads solution with 1.4×10^6^ beads/μL. After a 30-minute incubation at room temperature, the beads were washed with a magnetic separator, and an initial volume, i.e., 1-ethyl-3-(3-dimethylaminopropyl) carbodiimide + bead solution volume in the previous step, of 0.3 mg/mL ice cold solution of the capture antibody (UD1, UmanDiagnostics) was added. After a 2-hour incubation on a mixer (2,000 rpm, Multi-Tube Vortexer, Allsheng, China) at room temperature, the beads were washed, and an initial reaction volume of blocking solution was added. After 3 washes, the conjugated beads were suspended and stored at 4°C pending analysis. Before analysis, the beads were diluted to 2,500 beads/μL in bead diluent. The detection antibody (1 mg/mL, UD2, UmanDiagnostics) was biotinylated by adding 3% (vol/vol) 3.4 mmol/L EZ‐Link NHS‐PEG4‐Biotin (Quanterix), followed by a 30-minute incubation at room temperature. Free biotin was removed with spin filtration (Amicon Ultra-2, 50 kDa, Sigma, St. Louis, MO), and the biotinylated antibody was stored at 4°C pending analysis. The serum samples were assayed in duplicate on a Simoa HD-1 instrument (Quanterix) using a 2-step assay dilution protocol that starts with an aspiration of the bead diluent from 100 μL conjugated beads (2,500 beads/μL), followed by the addition of 20 μL biotinylated antibody (0.1 μg/mL) and 100 μL of 4-fold diluted sample (or undiluted calibrator) to the bead pellet. For both samples and calibrator, the same diluent was used (phosphate-buffered saline; 0.1% Tween-20; 2% bovine serum albumin; 10 μg/mL TRU Block [Meridian Life Science, Inc, Memphis, TN]). After a 47-cadence incubation (1 cadence = 45 seconds), the beads were washed, followed by the addition of 100 μL streptavidin-conjugated β-galactosidase (150 pmol/L, catalog No. 100439, Quanterix). This was followed by a 7-cadence incubation and a wash. Before reading, 25 μL resorufin β−D-galactopyranoside (catalog No. 100017, Quanterix) was added. The calibrator curve was constructed by use of the standard from the NfL ELISA (NF-Light, UmanDiagnostics) in triplicate. The lower limits of detection and quantification, as defined by the concentration derived from the signal of blank samples (sample diluent) + 3 and 10 SD, were 0.97 and 2.93 pg/mL, respectively. To evaluate the linearity of the assay, 6 different samples were analyzed at 4- (default), 8-, and 16-fold dilution, and the average coefficient of variation for the concentration measured at the different dilutions was 11.5%. All samples were measured as duplicates. The mean coefficient of variation of duplicate concentrations was 4.3%. In addition, a quality control sample was measured in duplicate on each of the 7 runs used to complete the study. The intra-assay coefficient of variation for this sample was <10%. All measurements were performed by board-certified laboratory technicians in one round of experiments using one batch of reagents.

### Psychometric assessment.

Forty-seven participants had psychometric testing at baseline, usually on the same day as serum sampling but at a maximum of 6 months from the time of sample collection (mean interval 0.0 years [SD 0.2 years]): 22 with bvFTD, 2 with FTD-MND, and 23 with PPA (9 with nfvPPA, 9 with svPPA, and 5 with PPA-NOS). Twenty-nine participants had follow-up psychometric testing at an interval of 1.1 years (SD 0.2 years): 11 with bvFTD, 2 with FTD-MND, and 16 with PPA (5 with nfvPPA, 7 with svPPA, and 4 with PPA-NOS). Testing included the Wechsler Abbreviated Scale of Intelligence Vocabulary, Block Design, Similarities, and Matrices subtests^20^; the Recognition Memory Tests for Faces and Words^21^; the Graded Naming Test^22^; the Graded Difficulty Calculation Test^23^; and the Delis-Kaplan Executive Function System Color-Word Interference Test,^24^ as well as the Mini-Mental State Examination.^25^

### Neuroimaging analysis.

Forty-six of the participants with FTD had volumetric T1 brain MRI on a 3T Siemens Trio scanner performed usually on the same day as serum sampling but at a maximum of 6 months from the time of sample collection (mean interval 0.0 years [SD 0.2 years]): 24 with bvFTD, 2 with FTD-MND, and 20 with PPA (8 with nfvPPA, 8 with svPPA, 4 with PPA-NOS). Twenty-nine participants had a follow-up scan at 1.1 years (SD 0.4 years) after the baseline scan: 13 with bvFTD, 2 with FTD-MND, and 14 with PPA (5 with nfvPPA, 6 with svPPA, and 3 with PPA-NOS). Whole-brain volumes were measured with a semiautomated segmentation method^26^ with annualized whole-brain atrophy rates calculated with the boundary shift integral.^27^ Individual lobar cortical volumes were measured with a multiatlas segmentation propagation approach following the brainCOLOR protocol (www.braincolor.org), combining regions of interest to calculate gray matter volumes for each lobe.^28,29^ Annualized lobar atrophy rates were calculated using the differences in volumes between the baseline and follow-up scans and dividing by the interval between scans.

### Statistical analysis.

Serum NfL concentrations were initially compared between the control group and the total FTD group. The Levene test for homogeneity demonstrated unequal variances between these 2 groups (Levene statistic = 22.8; *p* < 0.001); therefore, the Welch *t* test (without assumptions for equal variance) was used to compare the groups. Serum NfL data were normally distributed (Kolmogorov-Smirnov test), so an analysis of variance was used to compare mean serum NfL concentrations across each of the clinical subgroups (bvFTD, FTD-MND, nfvPPA, svPPA, lvPPA, and PPA-NOS) and across the genetic FTD subgroups (*MAPT*, *GRN*, and *C9orf72*), and to compare each of these subgroups with the control group. To allow for unequal variance, the Games-Howell correction was used for post hoc pairwise comparisons between groups. The same statistical methods were also used to compare NfL levels between the genetic subgroups and between each of these groups and the control group. The Pearson correlation coefficient was used to examine the association between serum NfL concentrations and each of the cognitive and imaging measures (with a Bonferroni correction for multiple comparisons also assessed, i.e., *p* < 0.005 for the cognitive measures and *p* < 0.007 for the imaging measures).

## RESULTS

**Figure 1:**
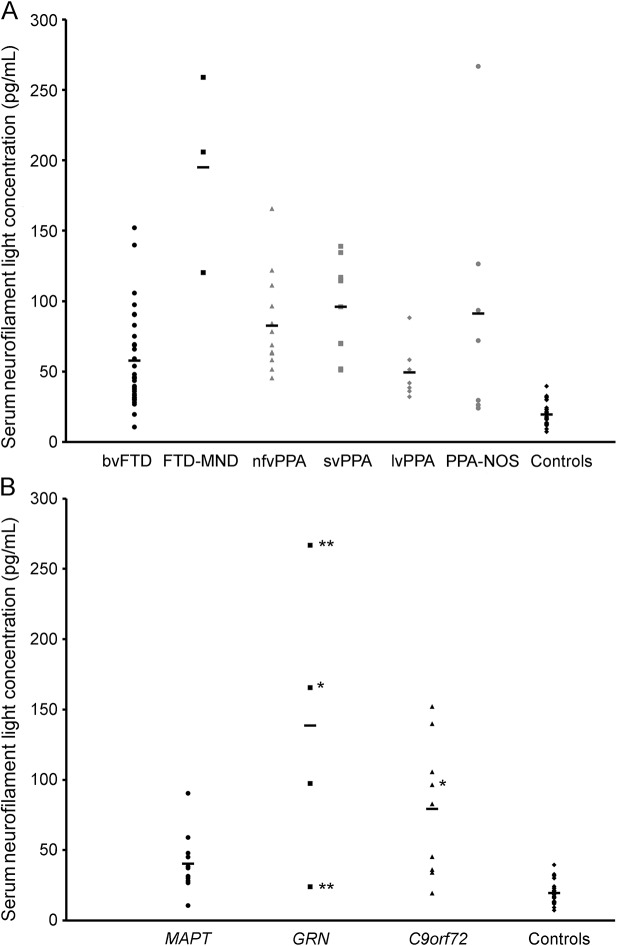
Serum neurofilament light chain concentrations in participants by (A) clinical diagnosis and (B) genetic status All genetic FTD patients have behavioral variant of frontotemporal dementia (bvFTD) except for those *with nonfluent variant of primary progressive aphasia (nfvPPA) and **with primary progressive aphasia not otherwise specified (PPA-NOS). FTD-MND = frontotemporal dementia– motor neuron disease; lvPPA = logopenic variant of primary progressive aphasia; svPPA = semantic variant of primary progressive aphasia.

Serum NfL concentrations in the control and total FTD groups and in each clinical subgroup are shown in table 1. The lowest serum NfL concentration in the study (7.2 pg/mL) was well above the lower limits of detection and quantification of the assay. Serum NfL concentrations were significantly higher in the total FTD group vs controls (mean 77.9 pg/mL [SD 51.3 pg/mL] and 19.6 pg/mL [SD 8.2 pg/mL] respectively; mean difference = 58.3 pg/mL, 95% confidence interval 45.4–71.1; *p* < 0.001). In distinguishing FTD from controls, a cutoff of 33 pg/mL gave a sensitivity of 84% and specificity of 96%. Serum NfL concentrations were also significantly higher in the majority of the clinical FTD subgroups compared with the control group (Welch statistic = 25.1, *df* 5, 13.2; *p* < 0.001) (figure 1A, table 2). Compared with controls, serum NfL concentrations were higher in patients with bvFTD, nfvPPA, and svPPA. Although patients with FTD-MND had higher mean serum NfL concentrations than controls (and all of the other groups), this difference did not reach statistical significance, likely because of the small sample size of the FTD-MND group. Serum NfL concentrations did not differ significantly between any of the clinical FTD subgroups, although there was a (not significant) trend toward a higher level in patients with svPPA compared with patients with bvFTD (mean difference = 38.1, *p* = 0.070). There was a significantly higher level in patients with svPPA compared with lvPPA (mean difference = 46.3, *p* = 0.032).

**Table 2:**
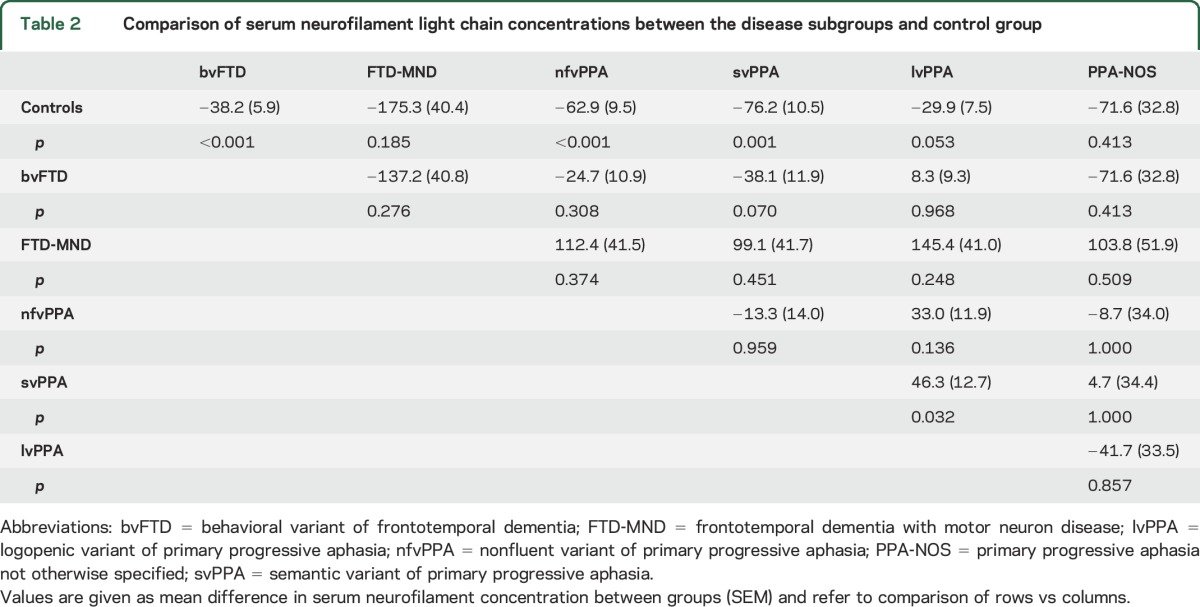
Comparison of serum neurofilament light chain concentrations between the disease subgroups and control group

Mean NfL concentrations were higher than controls in each of the genetic subgroups (figure 1B, table 3): 138.5 pg/mL (SD 103.3 pg/mL) in *GRN*, 79.2 pg/mL (SD 48.2 pg/mL) in *C9orf72*, and 40.5 pg/mL (SD 20.9 pg/mL) in *MAPT* mutations. However, only the *MAPT* subgroup (mean difference from controls = 20.8, 95% confidence interval = 1.4–40.3, *p* = 0.035) and the *C9orf72* subgroup (mean difference from controls = 59.5, 95% confidence interval = 8.0–111.0, *p* = 0.025) were significantly different, with the lack of difference in the *GRN* subgroup likely due to small sample size (table 3). Despite the apparent larger mean NfL levels in *GRN* and *C9orf72* compared with *MAPT* mutations, there was no significant difference in levels between the genetic subgroups (table 3).

**Table 3:**
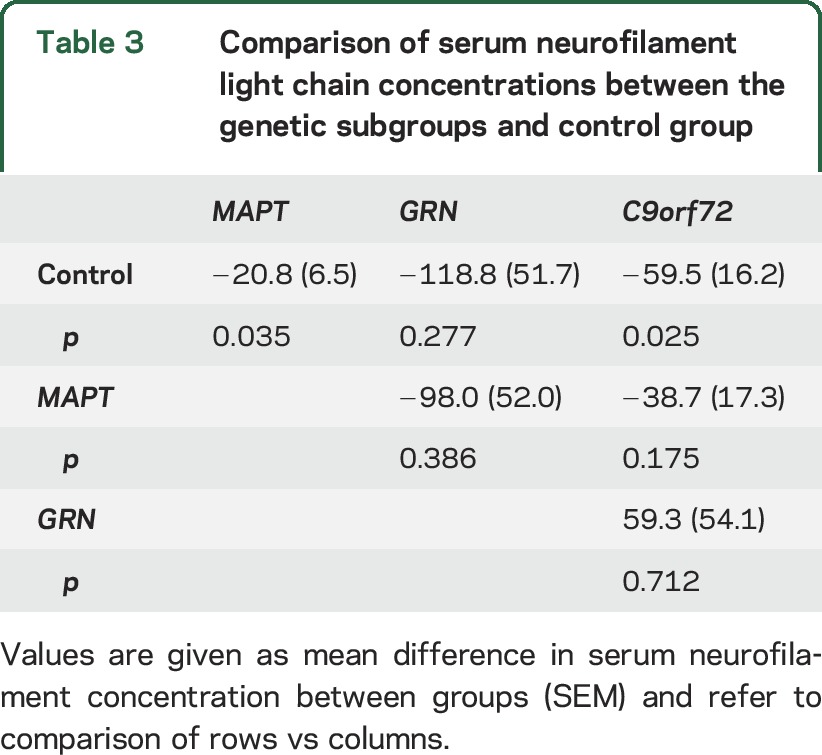
Comparison of serum neurofilament light chain concentrations between the genetic subgroups and control group

**Figure 2:**
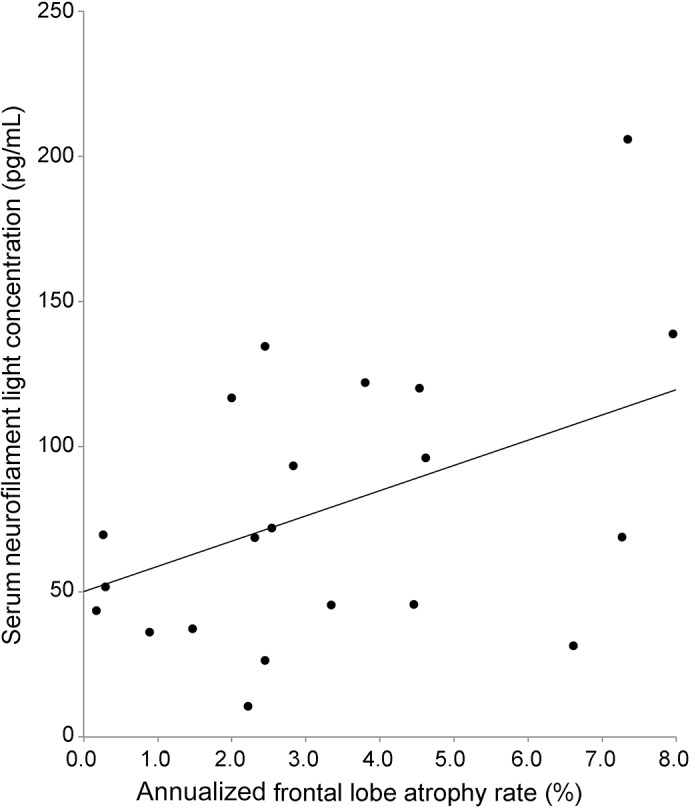
Relationship of serum neurofilament light chain (NfL) concentrations to frontal lobe atrophy rate Serum NfL concentrations are correlated with frontal lobe atrophy rates (*r* = 0.53, *p* = 0.003). Points indicate individual patient values, and the straight line indicates the line of best fit from a linear regression model of serum NfL on annualized frontal lobe atrophy rate.

Baseline and longitudinal cognitive and imaging measures are shown in table 4. Serum NfL concentrations correlated with baseline measures of executive dysfunction (Wechsler Abbreviated Scale of Intelligence similarities [*r* = −0.32, *p* = 0.03] and Delis-Kaplan Executive Function System Color-Word Interference ink color naming task [*r* = −0.35, *p* = 0.03]) but not with other baseline psychometric tests or with longitudinal changes in psychometric measures. However, no cognitive measures survived correction for multiple comparisons. There were also no significant correlations with baseline brain volumes. However, serum NfL levels were correlated with rates of whole brain (*r* = 0.46, *p* = 0.01), frontal lobe (*r* = 0.53, *p* = 0.003; figure 2), and parietal lobe (*r* = 0.38, *p* = 0.04) atrophy, although not with other lobar atrophy rates. Only the correlation with frontal lobe atrophy rate survived correction for multiple comparisons.

**Table 4:**
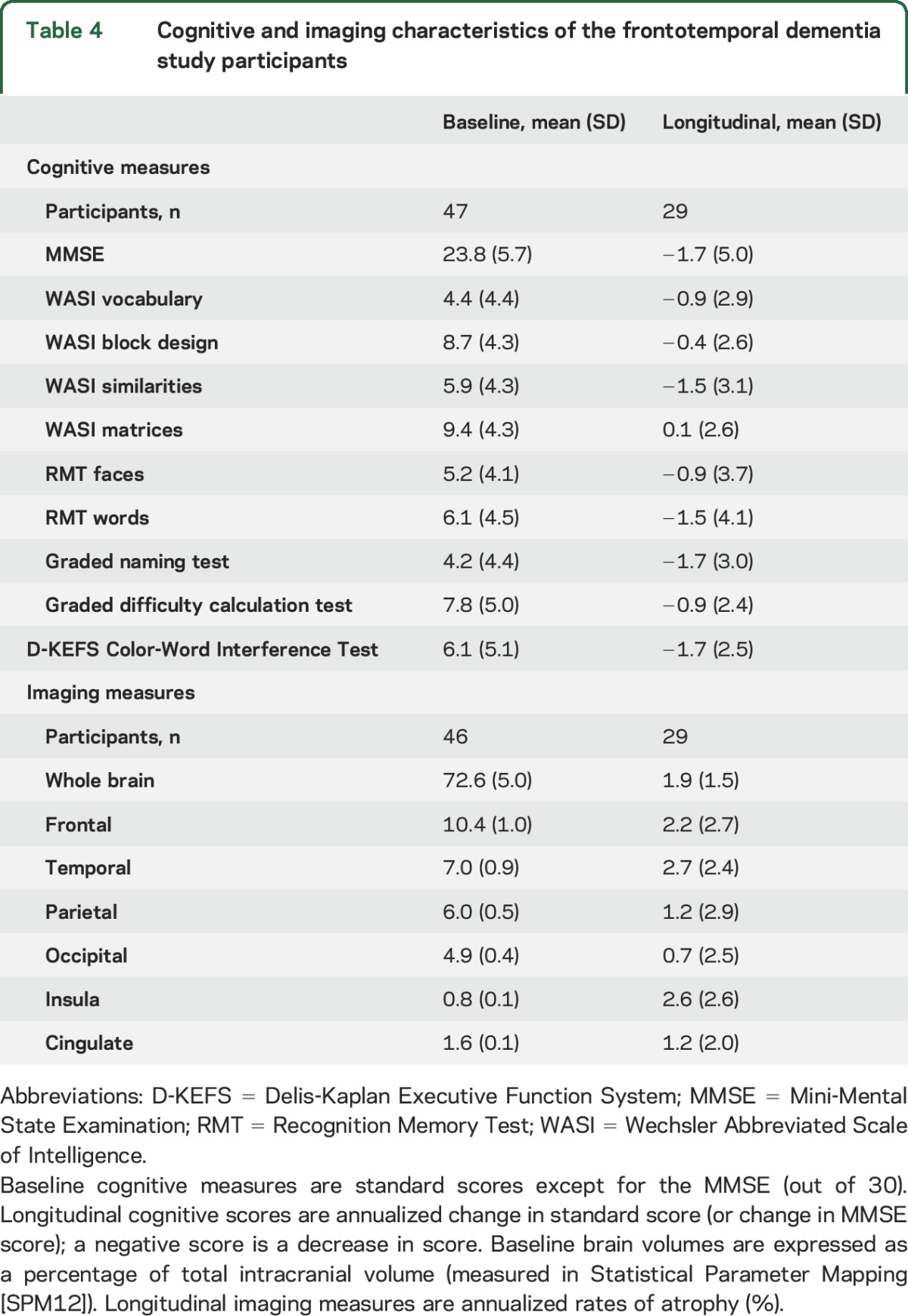
Cognitive and imaging characteristics of the frontotemporal dementia study participants

## DISCUSSION

Using an ultrasensitive immunoassay, we show that serum NfL concentrations are raised in FTD and that higher concentrations are associated with faster rates of brain atrophy. These findings suggest that serum NfL concentrations reflect the intensity of the disease in FTD and that higher concentrations are associated with a more rapid disease progression. Within the FTD subtypes, there was a tendency for groups with probable TDP-43 pathology (svPPA and FTD-MND clinically, *GRN* and *C9orf72* mutations genetically) to have raised levels compared with those associated with tau pathology (*MAPT* mutations), although within all groups there is substantial variability. With a lower limit of quantification of 0.26 pg/mL, all samples, including those from normal controls, could be reliably quantified, which is an advantage over earlier studies on serum NfL in other conditions.^14,30–32^

The results of this study are consistent with those found in previous CSF studies of NfL concentrations in FTD: levels are consistently higher in patients with FTD^4–11^ and tend to be increased in those with probable TDP-43 pathology.^10,11^ Certainly for genetic FTD, for which *GRN* and *C9orf72* mutations are associated with TDP-43 pathology, this is consistent with the more rapid progression (and shorter disease duration) seen in many patients within these 2 mutation groups (independent of clinical syndrome) compared with the relatively slower progression of patients with *MAPT* mutations (which is associated with tau pathology).^33^ One previous study also suggested a correlation of CSF NfL with measures of disease severity and, consistent with our study, showed an association of levels with frontal lobe atrophy.^11^

We found that serum NfL levels were correlated with the rate of subsequent brain atrophy but not with the baseline brain volumes. Measures of brain atrophy are likely to be better measures of the disease intensity than just a single cross-sectional measure of the whole-brain or lobar volumes, which reflect disease duration and normal variation as well as disease activity. Serum NfL levels correlated with baseline measures of executive function but not with longitudinal measures. A number of the patients had scored at near the floor on executive tasks at baseline; therefore, there is less ability to measure progression with such measures when assessed longitudinally.

It will be important to investigate patients at different stages of the disease because this may influence the association between NfL and rates of atrophy. The Genetic Frontotemporal Dementia Initiative (GENFI) study (www.genfi.org.uk) has recently shown that pathologic rates of brain atrophy appear to start up to 10 years before symptom onset but are variable between different genetic mutations.^29^ If serum NfL concentrations represent measures of disease intensity, then we would predict that levels would start to increase around 10 years before onset and may provide a useful noninvasive marker of proximity to symptom onset.

There are a number of limitations to this study. In particular, the majority of the patients did not have pathologic confirmation of the cause of their illness, and future studies should investigate serum NfL levels in different FTD pathologies. Although there is a relatively large number of cases for a study of a rare disorder like FTD, the individual numbers are small in each subgroup (particularly the FTD-MND group), and it would be useful for future studies to investigate larger groups of the individual clinical and genetic subtypes. Clinical measures of disease staging in FTD have only recently been designed (such as the Frontotemporal Lobar Degeneration-Clinical Dementia Rating^34^ and Frontotemporal Dementia Rating Scale^35^) and were not available in this cohort; it will be important for future studies to compare such measures with serum NfL levels.

Higher serum NfL concentrations are associated with more rapid brain atrophy and may therefore reflect disease intensity in FTD. Because blood sampling is less invasive and has better patient acceptability than lumbar puncture, serum NfL may provide important prognostic information and prove to be a useful outcome measure for clinical trials in FTD. However, further studies will be required to understand the factors affecting the variability in NfL concentration and to determine whether it can be a useful measure within individual patients.

## Supplementary Material

Accompanying Comment

## GLOSSARY

bvFTD: behavioral variant frontotemporal dementia
FTD: frontotemporal dementia
GENFI: Genetic Frontotemporal Dementia Initiative
lvPPA: logopenic variant of primary progressive aphasia
MND: motor neuron disease
NfL: neurofilament light chain
nfvPPA: nonfluent variant of primary progressive aphasia
PPA: primary progressive aphasia
PPA-NOS: primary progressive aphasia not otherwise specified
Simoa: single-molecule array
svPPA: semantic variant of primary progressive aphasia
