# Supplementary material for: Serum neurofilament light chain protein is a measure of disease intensity in frontotemporal dementia
Source: Neurology. 2016 Sep 27;87(13):1329–36. doi: 10.1212/WNL.0000000000003154 (PMC5047041; doi:10.1212/WNL.0000000000003154)
Supplement: Accompanying Comment [file supp_87_13_1329__index.html]

Serum neurofilament light chain protein is a measure of disease intensity in frontotemporal dementia — Accompanying Comment 

# Serum neurofilament light chain protein is a measure of disease intensity in frontotemporal dementia

## Accompanying Comment

**Neurology® data supplements are not copyedited before publication. Published editorials and translations have been copyedited.  
 © 2016 American Academy of Neurology.  
  
 Files in this Data Supplement:**

- Accompanying Comment - PDF
