# Supplementary material for: Serum neurofilament light chain protein is a measure of disease intensity in frontotemporal dementia
Source: Neurology. 2016 Sep 27;87(13):1329–36. doi: 10.1212/WNL.0000000000003154 (PMC5047041; doi:10.1212/WNL.0000000000003154)
Supplement: Accompanying Comment [file supp_WNL.0000000000003154_WNL.0000000000003164v1.pdf]

7. Petzold A, Keir G, Warren J, Fox N, Rossor MN. A systematic review and meta-analysis of CSF neurofilament protein levels as biomarkers in dementia. Neurodegener Dis 2007;4:185–194.
8. Pijnenburg YA, Janssen JC, Schoonenboom NS, et al. CSF neurofilaments in frontotemporal dementia compared with early onset Alzheimer's disease and controls. Dement Geriatr Cogn Disord 2007;23:225–230.
9. de Jong D, Jansen RW, Pijnenburg YA, et al. CSF neurofilament proteins in the differential diagnosis of dementia. J Neurol Neurosurg Psychiatry 2007;78:936–938.
10. Landqvist Waldö M, Frizell Santillo A, Passant U, et al. Cerebrospinal fluid neurofilament light chain protein levels in subtypes of frontotemporal dementia. BMC Neurol 2013;13:54.
11. Scherling CS, Hall T, Berisha F, et al. Cerebrospinal fluid neurofilament concentration reflects disease severity in frontotemporal degeneration. Ann Neurol 2014;75:116–126.
12. Teunissen CE, Dijkstra C, Polman C. Biological markers in CSF and blood for axonal degeneration in multiple sclerosis. Lancet Neurol 2005;4:32–41.
13. Tortelli R, Ruggieri M, Cortese R, et al. Elevated cerebrospinal fluid neurofilament light levels in patients with amyotrophic lateral sclerosis: a possible marker of disease severity and progression. Eur J Neurol 2012;19:1561–1567.
14. Lu CH, Macdonald-Wallis C, Gray E, et al. Neurofilament light chain: a prognostic biomarker in amyotrophic lateral sclerosis. Neurology 2015;84:2247–2257.
15. Rissin DM, Kan CW, Campbell TG, et al. Single-molecule enzyme-linked immunosorbent assay detects serum proteins at subfemtomolar concentrations. Nat Biotechnol 2010;28: 595–599.
16. Kuhle J, Barro C, Andreasson U, et al. Comparison of three analytical platforms for quantification of the neurofilament light chain in blood samples: ELISA, electrochemiluminescence immunoassay and Simoa. Clin Chem Lab Med Epub 2016 Apr 12. doi: 10.1515/cclm-2015-1195.
17. Rascovsky K, Hodges JR, Knopman D, et al. Sensitivity of revised diagnostic criteria for the behavioural variant of frontotemporal dementia. Brain 2011;134:2456–2477.
18. Strong MJ, Grace GM, Freedman M, et al. Consensus criteria for the diagnosis of frontotemporal cognitive and behavioural syndromes in amyotrophic lateral sclerosis. Amyotroph Lateral Scler 2009;10:131–146.
19. Gorno-Tempini ML, Hillis AE, Weintraub S, et al. Classification of primary progressive aphasia and its variants. Neurology 2011;76:1006–1014.
20. Wechsler D. Wechsler Abbreviated Scale of Intelligence. San Antonio, TX: The Psychological Corporation; 1999.
21. Warrington EK. Manual for the Recognition Memory Test for Words and Faces. Windsor, UK: NFER-Nelson; 1984.
22. McKenna P, Warrington EK. Testing for nominal dysphasia. J Neurol Neurosurg Psychiatry 1980;43:781–788.
23. Jackson M, Warrington EK. Arithmetic skills in patients with unilateral cerebral lesions. Cortex 1986;22:611–620.
24. Delis DC, Kaplan E, Kramer JH. Delis-Kaplan Executive Function System (D-kefs). San Antonio, TX: The Psychological Corporation; 2001.
25. Folstein M, Folstein S, McHugh P. The "Mini Mental State": a practical method for grading the cognitive state of patients for the clinician. J Psychiatr Res 1975;12:189–198.
26. Freeborough PA, Fox NC, Kitney RI. Interactive algorithms for the segmentation and quantitation of 3-D MRI brain scans. Comput Methods Programs Biomed 1997;53:15–25.
27. Freeborough PA, Fox NC. The boundary shift integral: an accurate and robust measure of cerebral volume changes from registered repeat MRI. IEEE Trans Med Imaging 1997;16:623–629.
28. Cardoso MJ, Modat M, Wolz R, et al. Geodesic information flows: spatially-variant graphs and their application to segmentation and fusion. IEEE Trans Med Imaging 2015; 34:1976–1988.
29. Rohrer JD, Nicholas JM, Cash DM, et al. Presymptomatic cognitive and neuroanatomical changes in genetic frontotemporal dementia in the Genetic Frontotemporal dementia Initiative (GENFI) study: a cross-sectional analysis. Lancet Neurol 2015;14:253–262.
30. Disanto G, Adiutori R, Dobson R, et al. Serum neurofilament light chain levels are increased in patients with a clinically isolated syndrome. J Neurol Neurosurg Psychiatry 2016;87:126–129.
31. Kuhle J, Gaiottino J, Leppert D, et al. Serum neurofilament light chain is a biomarker of human spinal cord injury

Comment: "If you can't measure it, you can't improve it" (Lord Kelvin)

In clinical practice, disability and its progression are notoriously difficult to quantify, urging the need for reliable soluble biomarkers for neuroaxonal damage. Neurofilament light chain (NfL) is a scaffolding protein of the neural cytoskeleton with important roles in axonal and dendritic branching and growth. After neuronal damage, NfL levels in the CSF increase and thus are considered a highly specific, real-time biomarker of axonal injury.

In the case of serum NfL, the single-molecule array technology substantially improves analytical sensitivity to an extent that measurements in blood-derived samples may be used as surrogate endpoints in neuroprotection trials or even in daily practice in the relatively near future.[1,2] The ultrasensitive assay used by Rohrer and colleagues[1] allowed reliable NfL measurements in all serum samples, including healthy controls, a giant stride for a biomarker that is independent of CSF and hence applicable in a routine clinical setting. Moreover, the authors found that their assay is quantitative: higher concentrations were associated with more rapid course of frontal lobe atrophy ($r = 0.53$, $p = 0.003$).[1] A strong correlation between CSF and blood NfL levels has been convincingly shown across a number of neurologic diseases, so the lack of CSF data in the current study does not limit the relevance of the findings.

Rohrer and colleagues[1] provide strong evidence supporting the potential role of serum NfL as a disease progression marker in frontotemporal dementia. Their results are well in line with recent findings in experimental mouse models of proteopathic (tau, β-amyloid, and α-synuclein) neurodegenerative diseases in which NfL levels in CSF and plasma were responsive to experimental manipulation or targeted therapy.[3] Future studies in large, well-characterized patient cohorts are warranted to further characterize NfL as a primary biomarker to predict and monitor disease progression and to assess treatment responses.

1. Rohrer JD, Woollacott IOC, Dick KM, et al. Serum neurofilament light chain protein is a measure of disease intensity in frontotemporal dementia. Neurology 2016;87: 1329–1336.
2. Kuhle J, Barro C, Andreasson U, et al. Comparison of three analytical platforms for quantification of the neurofilament light chain in blood samples: ELISA, electrochemiluminescence immunoassay and Simoa. Clin Chem Lab Med Epub 2016 Apr 12. doi: 10.1515/cclm-2015-1195.
3. Bacioglu M, Maia L, Preische O, et al. Neurofilament light chain in blood and CSF as marker of disease progression in mouse models and in neurodegenerative diseases. Neuron 2016;91:56–66.

Jens Kuhle, MD, PhD

From Neurology, Departments of Medicine, Clinical Research and Biomedicine, University Hospital Basel, Switzerland.
Study funding: No targeted funding reported.
Disclosure: J.K. reports that the University Hospital Basel as the employer of J.K. has received and dedicated to research support consulting fees from Novartis and Protagen AG; speaker fees from the Swiss MS Society, Biogen, Novartis, Roche, and Genzyme; travel expenses from Merck Serono and Novartis; grants from ECTRIMS Research Fellowship Programme, University of Basel, Swiss MS Society, Swiss National Research Foundation, Bayer (Schweiz) AG, Genzyme, Novartis, and Roche. Go to Neurology.org for full disclosures.

© 2016 American Academy of Neurology. Unauthorized reproduction of this article is prohibited.
